
JOURNAL INFORMATION
==============================
NLM Title Abbreviation: Behav Neurol
Journal ID: Behav Neurol
NLM Journal ID: 8914585
Journal ID: BN

Behavioural Neurology

ISSN: 0953-4180
EISSN: 1875-8584
Publisher: Wiley

ARTICLE INFORMATION
==============================
PMCID: PMC5215760
PMID: 23187146
DOI: 10.3233/BEN-120314
Article ID: 262047
Article version: 1
Subjects: Editorial

Researching Tourette Syndrome in Europe

Cavanna Andrea Eugenio 1 2 *
1 The Michael Trimble Neuropsychiatry Research Group Department of Neuropsychiatry BSMHFT and University of Birmingham Birmingham UK
2 Department of Motor Neuroscience and Movement Disorders Institute of Neurology and University College London London UK
*Andrea Eugenio Cavanna: a.cavanna@ion.ucl.ac.uk
Print publication date: 2013
Volume: 27
Issue: 1
First page: 1
Last page: 2
Received 2012 Nov 23; Accepted 2012 Nov 23
Copyright: Copyright © 2013 Hindawi Publishing Corporation and the authors.
Copyright year: 2013
License: This is an open access article distributed under the Creative Commons Attribution License, which permits unrestricted use, distribution, and reproduction in any medium, provided the original work is properly cited.
License URL: https://creativecommons.org/licenses/by/3.0/

==============================
Behavioural Neurology 27 (2013) 1–2
DOI 10.3233/BEN-120314
IOS Press

### Editorial

### Researching Tourette syndrome in Europe

Andrea Eugenio Cavanna[a,b,*]
[a] *The Michael Trimble Neuropsychiatry Research Group, Department of Neuropsychiatry, BSMHFT and University of Birmingham, Birmingham, UK*
[b] *Department of Motor Neuroscience and Movement Disorders, Institute of Neurology and University College London, London, UK*

Gilles de la Tourette syndrome was first described in Europe by the Parisian school of Jean-Martin Charcot in the second half of the XIX century. In 1885, French physician Georges Albert Edouard Brutus Gilles de la Tourette (1857–1904), one of the most pre-eminent figures in Charcot's neuropsychiatry school, published a case series of nine patients who presented with the clinical triad of tics, echolalia and coprolalia. It was Charcot himself who named the condition after Gilles de la Tourette, securing eponymic (hence immortal) fame to his flamboyant pupil.

As a medical condition, Gilles de la Tourette syndrome has a fascinating history on its own. Long considered to be a rare, somewhat bizarre medical curiosity, it progressively shifted towards the concept of a relatively common disorder, which in its milder forms can be regarded as a variant of healthy human behaviour. Recent estimates suggested that the prevalence of Tourette syndrome (or TS – as it is now called, especially in the American literature) can be as high as 1% in school-age children, if diagnosed according to current diagnostic criteria. Most importantly, TS' unique position at the interface between neurology and psychiatry has attracted the interest of scholars and researchers, which devote their efforts to improve our understanding of the causes and effects of this fascinating condition from both clinical and scientific perspectives.

This thematic issue covers the state of the art of European research on TS, which went through a renaissance throughout the last decade, and gained momentum in joining the scientific efforts of the USA-Tourette Syndrome Association, which had already been established since 1972. The introductory article by Rickards et al. outlines the history of the young European Society for the Study of Tourette Syndrome (ESSTS), including antecedents and current trends.

Charcot himself first characterised TS as being associated with behavioural symptoms, including obsessive compulsive symptoms (which he defined as "des idées fixes") and provided the example of one of his patients, "not being able to open the door without first turning the door knob three or four times, or saying out loud 'one, two, three, four'…". The paper by Debes et al. focuses on the behavioural spectrum of TS, which extends to attention deficit and hyperactivity disorder, affective disorders and impulse control disorders. Although the majority of patients with TS do not present with significant cognitive problems, specific alterations in social cognition have recently been identified and linked with underlying fronto-striatal dysfunction, as revealed in the contribution by Eddy and Cavanna. The exact mechanisms responsible for the pathophysiology are still unknown and intensely researched: the possible role of immune dysfunction in the pathogenesis of TS is addressed by Elamin et al., whilst Eichele and Plessen review intriguing findings of neural plasticity as revealed by functional and struc-

*Corresponding author: Prof Andrea Eugenio Cavanna, MD, PhD, Department of Neuropsychiatry, The Barberry National Centre for Mental Health, Birmingham B15 2FG, UK. Tel.: +44 121 3012317; Fax: +44 121 3012291; E-mail: a.cavanna@ion.ucl.ac.uk.

ISSN 0953-4180/13/$27.50 © 2013 – IOS Press and the authors. All rights reserved
This article is published online with Open Access and distributed under the terms of the Creative Commons Attribution Non-Commercial License.

tural magnetic resonance imaging studies. Sensorimotor networks have been extensively investigated in patients with TS: in this special issue, the article by Rothenberger and Roessner focuses on functional neuroimaging investigations, whereas the contribution by Orth and Münchau covers novel findings from transcranial magnetic stimulation studies. Rajagopal et al. explore the possible link between alterations in sensorimotor processing and specific experiences of 'premonitory urges' to tic, which are often reported by patients with TS. Little is known about another important aspect of the pathophysiology of TS, i.e. the mechanisms which maintain tic behaviours: the article by Beetsma et al. presents the findings of an original study addressing this topic.

TS can have a profound impact on patients' overall wellbeing through its physical, psychological and social effects. The contributions by Cavanna et al. review the first decade of research on the determinants of health-related quality of life in TS and present the development and validation of the first disease-specific quality of life instrument for young patients with TS (C&A-GTS-QOL). Over the last decade, it has become clear that the treatment of TS, which starts from psychoeducation, relies on three main components: behavioural therapy (reviewed in the paper by Frank and Cavanna), pharmacotherapy (including off-license indications: article on cannabinoids by Müller-Vahl) and functional neurosurgery (deep brain stimulation: contributions by Porta et al. and Ackermans et al.). This special issues closes with an outline of the possible trajectories for future research on TS (Cavanna et al.).

The website of the Italian Association on TS welcomes its readers with an interesting quotation from the correspondence between two champions of the modern sciences of the mind, Russian neuropsychologist and developmental psychologist Alexander Luria and British neurologist and biologist Oliver Sacks. This quotation is also a measure of the relevance of TS to the field of behavioural neurology: according to Luria, our understanding of TS will improve, necessarily and by large, our understanding of human nature in general, as he is not aware of any other syndrome of similar interest. In addition to an updated educational tool on TS by cutting-edge European academic groups, the present volume provides a tribute to clinicians and researchers of all times who could not resist the fascination of this quintessentially neurobehavioural disorder. Finally, the editor of this special issue of Behavioural Neurology wishes to dedicate this collaborative effort to all those young and adult patients, who fight ignorance and prejudice on TS throughout their lives.
